# Fetal Brain Elicits Sexually Conflicting Transcriptional Response to the Ablation of Uterine Forkhead Box A2 (*Foxa2*) in Mice

**DOI:** 10.3390/ijms22189693

**Published:** 2021-09-07

**Authors:** Pramod Dhakal, Monica Strawn, Ananya Samal, Susanta K. Behura

**Affiliations:** 1 Division of Animal Sciences, University of Missouri, 920 East Campus Drive, Columbia, MO 65211, USA; dhakalp@missouri.edu (P.D.); mpsrkf@mail.missouri.edu (M.S.); as4w5@missouri.edu (A.S.); 2 MU Institute for Data Science and Informatics, University of Missouri, Columbia, MO 65211, USA

**Keywords:** fetal brain, sexual conflict, longevity, placenta, uterus

## Abstract

In this study, we investigated the effects of ablation of uterine Forkhead Box A2 (*Foxa2*) on gene expression of fetal brain relative to placenta. Using a conditional knockout mouse model for uterine *Foxa2*, here we show that the lack of uterine *Foxa2* elicits a sexually-conflicting transcriptional response in the fetal brain relative to placenta. The ablation of *Foxa2* in the uterus altered expression of genes related to growth, nutrient sensing, aging, longevity and angiogenesis among others. In the wildtype mice, these genes were expressed higher in the fetal brain and placenta of males compared to females. However, in mice lacking uterine *Foxa2*, the same genes showed the opposite pattern i.e., higher expression in the fetal brain and placenta of females compared to males. Based on the known marker genes of mice placenta and fetal brain cells, we further predicted that the genes exhibiting the sexually conflicting expression were associated with vascular endothelial cells. Overall, our study suggests that uterine *Foxa2* plays a role in the regulation of the brain-placental axis by influencing the fetoplacental vascular changes during pregnancy.

## 1. Introduction

The Forkhead box A2 (*Foxa2*) is a major transcription factor that plays diverse roles in reproduction, development and also human diseases [1]. It is required to regulate the processes associated with gastrulation [2], neural tube patterning [3,4], notochord morphogenesis, and patterning of dorsal foregut endoderm [2,5]. The role of *Foxa2* in uterine gland development and pregnancy establishment has been studied [6]. *Foxa2* is necessary for female fertility in mice and humans [7,8]. Mice lacking uterine *Foxa2* are infertile, but pregnancy can be rescued by injection of leukemia inhibitory factor (LIF) [8,9]. LIF is expressed in the uterus in response to estrogen from the ovary, and requires the transcription factor *Foxa2* for blastocyst implantation [6,7]. The conditional knockout of *Foxa2* in the uterus alters gene expression of the placenta [10]. Moreover, there is remarkable coordination in the gene expression between the placenta and fetal brain, suggesting a functional interaction between the brain and placenta (aka the brain-placental axis) [10]. The brain-placental axis, however, is vulnerable to intrauterine conditions that influence pregnancy outcomes [11].

The placenta plays indispensable roles in the development of fetus. Emerging evidences show that placenta plays important roles in the developmental process of the brain [5,6,7,8]. Genesis of the neural tube that eventually leads to the development of the central nervous system (CNS) occurs during gestation days 9–9.5 in mice [9]. Placental dysfunction can lead to defective neuronal development that causes different brain disorders in early childhood and later in life in humans [12]. However, placental development and function are differentially modulated based on fetal sex. In rodents, the placenta develops at a relative faster rate in males compared to females [13]. Relationships between fetal sex and placental functions have been demonstrated in many studies [14,15,16,17,18,19]. Recent study shows that uterine *Foxa2* influences the growth of fetus and placenta in a sexual dimorphic manner [20]. The current study addresses the questions as to whether a uterus lacking *Foxa2* has an influence on the transcriptional response of the fetal brain, and whether that influence is conditioned by the fetal sex and placenta.

## 2. Results

### 2.1. Lack of Uterine Foxa2 Altered Fetal and Placental Weight in a Sex-Biased Manner

**Figure 1:**
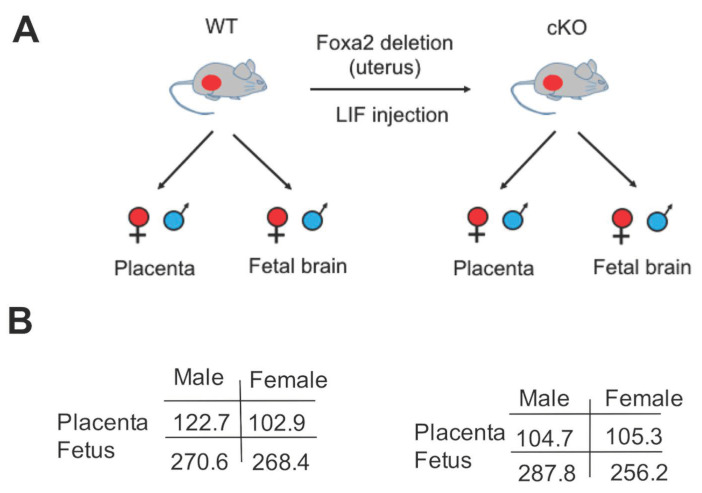
Overview of the study. (**A**) Experimental design. The WT and *Foxa2* cKO pregnant mice (red dot indicating pregnant) were dissected to collect fetal brain and placenta samples of both sexes. (**B**) Comparison of fetal and placental weight. M: male and F: female. Weights, in milligram, of the fetus and placenta of the WT (**left**) and cKO mice (**right**). Sample size: n = 24.

The weights of male and female fetuses and their corresponding placentae of the wildtype (WT) and conditional knockout (cKO) mice were measured on the gestation day 15 (GD15) (Figure 1A). In the WT mice, the mean weight of the male fetuses was only ~2 mg more than that of the female fetuses. But the mean weight of placentae was ~20 mg more for males compared to females (Figure 1B). In the cKO mice, a contrasting growth pattern was observed. In those mice, the male fetuses weighed ~30 mg heavier than the female fetuses, but the placental weight was only 0.6 mg different. Although the sex difference in the weight of placenta and fetal brain was not statistically significant (*p* = 0.36), the fetoplacental growth was dysregulated due to the absence of *Foxa2* in the uterus in a consistent manner that was found at other gestation times in mice [20].

### 2.2. Global Alteration in Gene Expression of Fetal Brain and Placenta in Response to the Ablation of Uterine Foxa2

**Figure 2:**
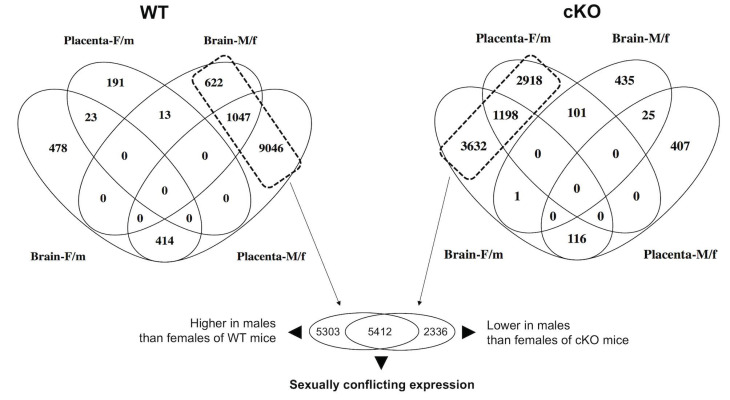
Venn diagram showing the number of genes expressed in a sex-biased manner in the placenta and fetal brain of the WT and cKO mice (sample size: n = 24). The male-biased expression is shown as M/f and the female-biased expression is shown as F/m. The dotted rectangle shows that the majority of the genes are expressed in a sex-biased manner in the WT and *Foxa2* cKO mice. They include 5412 common genes that showed sexually conflicting expression between placenta and fetal brain when the uterus lacked *Foxa2*.

RNA-seq showed widespread dysregulation of genes in the placenta and fetal brain due to the lack of *Foxa2* in the uterus. In the WT mice, the majority of the genes showed a male-biased expression pattern in the fetal brain and placenta (Figure 2). The placenta showed 9046 genes that were expressed in a male-biased manner in the WT mice. When the placenta and fetal brain were considered, a total of 10,715 (622 + 1047 + 9046) genes were expressed in a male-biased manner (see Venn diagram in Figure 2). But, only 692 (191 + 23 + 478) genes were expressed in a female-biased manner in those mice. In the cKO mice, 867 (435 + 25 + 407) genes were expressed in a male-biased manner whereas 7748 (2918 + 1198 + 3632) genes were expressed in a female-biased manner. This pattern showed a significant bias (Chi Square 14,158.5, *p* < 0.0001) in gene expression between male and female fetuses of the WT vs. cKO mice. The fold change in number of female-biased genes was 11.19 increase, and the fold change in the number of male-biased genes was 12.35 decrease in the cKO mice relative to the WT mice. The genes showing sex-biased expression in the WT and cKO mice are listed in Table S1 and Table S2 respectively.

When we compared the male-biased genes of WT with the female-biased genes of cKO mice (see the dotted boxes in Figure 2), thousands of genes (total 5412 genes) were found common. These genes are listed in Table S3. Each of those 5412 genes was expressed at a higher level (fold changes varied from 1.1 to 45.1) in the male fetal brain and placenta of the WT mice. In mice lacking uterine *Foxa2*, the same genes showed higher level (fold changes varied from 1.12 to 54.4) of expression in the female fetal brain and placenta (Figure 2). This change in the sex-biased gene expression pattern- from male-biased in WT to female biased in cKO- suggested that fetal brain elicited sexually conflicting transcriptional response to the placenta due to the absence of uterine *Foxa2*.

### 2.3. Lack of Uterine Foxa2 Altered Growth and Nutrient Signaling Genes in the Placenta and Fetal Brain

**Figure 3:**
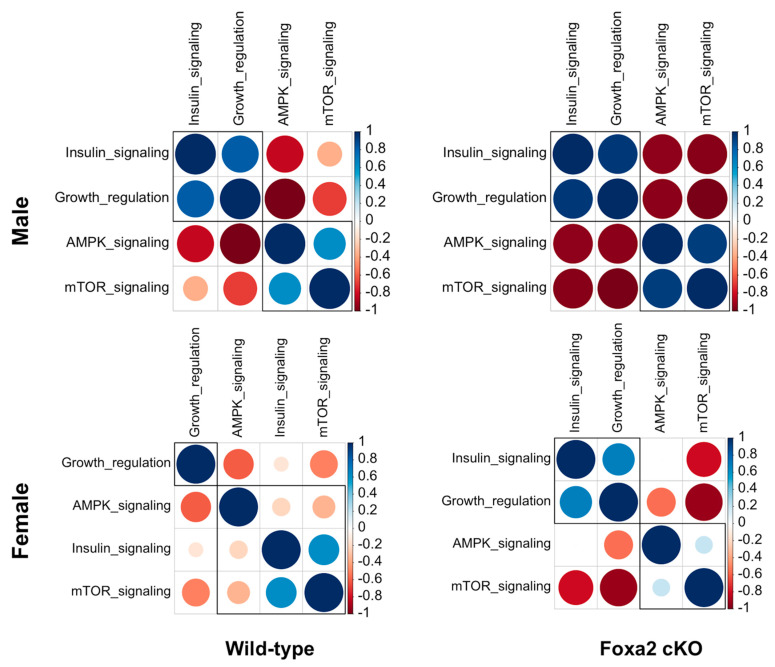
Pair-wise correlation of expression of genes related to the regulation of growth and nutrient sensing and signaling pathways in the brain and placenta male versus female fetuses of the WT and cKO mice (sample size: n = 24). The hierarchical cluster patterns are shown by box. The color-coded scale of correlation levels is shown to the right of each plot.

We performed functional annotation of the genes (n = 5412) that showed sexually conflicting expression between placenta and fetal brain in response to uterine ablation of *Foxa2.* GO enrichment analysis identified significant over-representation (Fisher Exact test, *p* < 0.05) of regulation of growth, and the signaling pathways of insulin growth factor 1 (IGF-1), mechanistic target of rapamycin (mTOR), and adenosine monophosphate-activated protein kinase (AMPK) among those genes (Table S4). The IGF-1, mTOR and AMPK pathways are known to regulate nutrient sensing and signaling during reproduction and development [21,22,23,24,25]. Pair-wise correlation analysis was performed with gene expression variation of genes associated with these pathways (Figure 3). It showed differential correlation of growth regulation with insulin, AMPK and mTOR signaling pathways in male versus female fetuses of the WT and cKO mice.

### 2.4. Differential Expression of Longevity Genes in the Placenta and Fetal Brain in Response to the Lack of Uterine Foxa2

**Figure 4:**
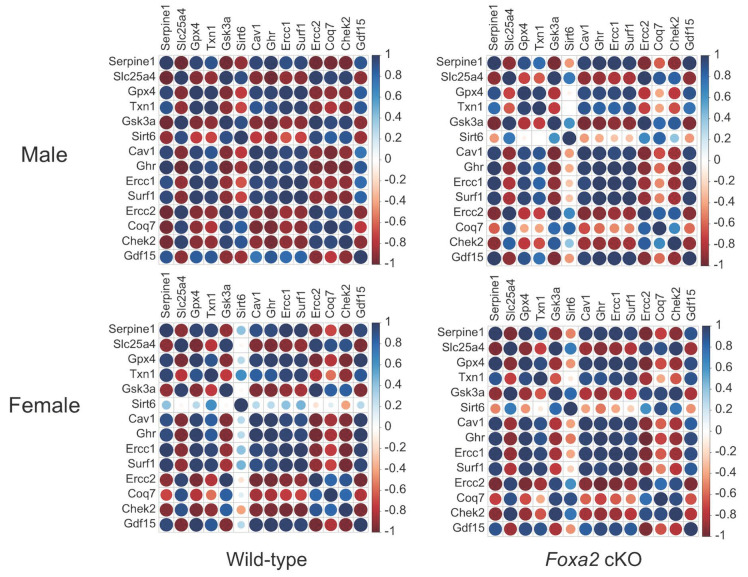
Correlation plots showing expression of *Sirt6* relative to the expression of other longevity genes in the WT versus cKO mice of both sexes (sample size: n = 24). The color-coded scale of correlation levels is shown on the right to each plot.

**Figure 5:**
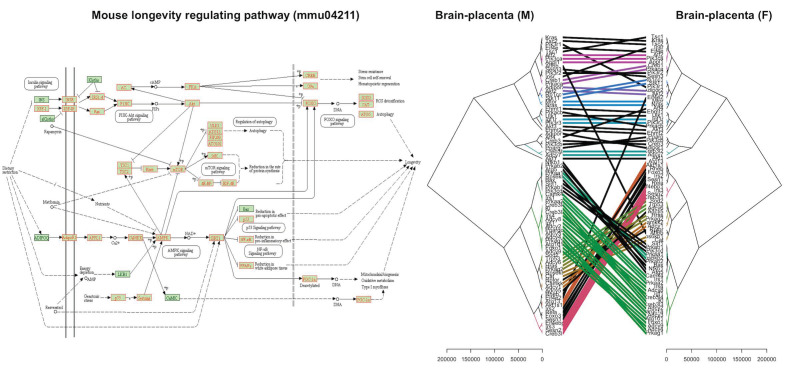
(**Left**): The mouse longevity regulation pathway (Kyoto Encyclopedia of Genes and Genomes, KEGG, pathway# mmu04211) genes expressed in the fetal brain and placenta are shown in red. (**Right**): A tanglegram plot shows the expression pattern of the longevity regulation pathway genes in the placenta and fetal brain of males vs. females (sample size: n = 24). The scales below the plots show the measure of branch heights of the respective cladograms.

We identified specific pro-longevity and anti-longevity genes that were differentially expressed in the fetal brain and placenta in response to the ablation of uterine *Foxa2* (Table S5). The longevity function of those genes was demonstrated in earlier studies, and curated in the mouse GeneAge database [26]. Our analysis showed that *Sirt6* that controls mouse longevity [27] was altered in a sex-dependent manner due to the ablation of uterine *Foxa2*. In the WT mice, *Sirt6* showed a higher correlation in expression with other longevity genes (Figure 4) in the males compared to females. We didn’t observe the same pattern in the cKO mice. It is known that *Sirt6* controls mouse longevity in males only [27]. In addition, we observed that the mouse longevity regulation pathway (KEGG pathway # mmu04211) was dysregulated due to the lack of uterine *Foxa2* (Figure 5). More than 93% (84 out of 90) of the genes associated with this pathway showed altered expression in the placenta and fetal brain when the uterus lacked *Foxa2*. Comparative cluster analysis of fold-changes in gene expression (Figure 5) showed that the longevity regulation pathway was affected differentially between males and females due to the absence of *Foxa2* in the uterus.

### 2.5. Angiogenesis Genes Were Affected in the Fetal Brain and Placenta due to the Lack of Uterine Foxa2

**Figure 6:**
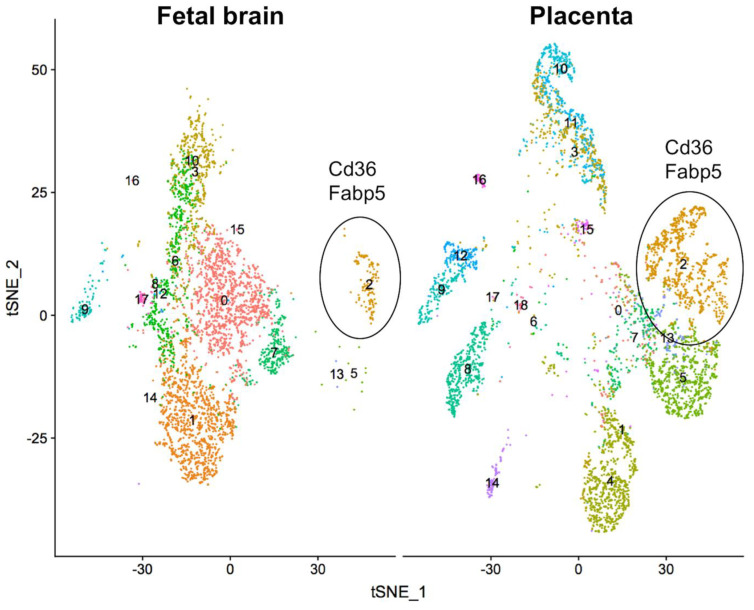
Meta-analysis of DEGs of placenta and fetal brain samples (n = 24) from the current study in comparison with the marker genes from a previously published single-cell expression data of mouse fetal brain and placenta [28]. It showed canonical correlation in gene expression in different clusters of cells (numbered) between fetal brain and placenta. The cluster #2 was identified as endothelial cells based on marker genes *CD36* and *Fabp5*.

We identified several angiogenesis-related genes that were altered in the placenta and fetal brain of mice lacking uterine *Foxa2* compared to the WT mice (Table S6). We made use of the single-cell gene expression data of mouse placenta and fetal brain from an earlier study [28] to determine if these angiogenesis genes are markers of specific cell types of the placenta and fetal brain. We downloaded the single-cell expression data, and identified cells (see Methods) that expressed genes in a canonically correlated manner between the placenta and fetal brain (Figure 6). A specific cluster of cells (cluster #2, see Figure 6) was predicted as vascular endothelial cells based on the expression of marker genes *Cd36* and *Fabp5.* The cells within this specific cluster expressed additional genes (n = 169) whose expression level was significantly different compared to expression in other cell clusters (Table S7). Each of those marker genes showed a sex-biased expression pattern in the brain and placenta (Table S7) in our RNA-seq data. These findings suggested that the lack of *Foxa2* in the uterus impacted gene expression of the vascular endothelial cells of the placenta and fetal brain.

## 3. Discussion

Fetal development is intricately linked to the development of placenta [29]. In mice, the fetal and placental weights change in a correlated manner during mid-gestation [30]. The growth of the placenta is also dependent upon the fetal sex. The male placenta weighs ~12% heavier than female placenta in rats on GD15 [13]. We observed that GD15 placenta had higher weight in males than females in the WT mice. The fetal weight was marginally different (~2 mg) between sexes in those mice. In mice lacking the uterine *Foxa2*, the fetus showed higher weight in males compared to females. However, the placenta weight was only 0.6 mg different between males and females. This finding agreed that *Foxa2* has a sex-biased effect on fetoplacental development in mice [20], but further suggested that uterine ablation of *Foxa2* triggered a trade-off effect, which refers to a condition where one trait benefits at the cost of another [31], between placental and fetal development. This is consistent with the idea that fitness for growth and for reproduction can influence each other [32]. Our data showed a change in expression of as many as 5412 genes between the placenta and fetal brain of males and females. It is further known that sex-biased gene expression is required to maintain a balance between sex-specific fitness in reproduction and development [33].

Our data showed sex-biased expression of 199 marker genes of vascular endothelial cells (Table S6), many related to angiogenesis, suggesting that placental endothelia may play a key role in sex-specific fetoplacental communication. The vascular endothelial cells play important roles in the regulation of placental barrier [34] and fetoplacental communication [35]. In the brain, vascular endothelial cells constitute a major component of the neurovascular unit and choroid plexus. The neurovascular endothelial cells interact with the local neuronal circuits, glia, and pericytes to control blood flow and regulate the blood-brain barrier [36]. The placental vascular endothelial cells constitute a major component of the placental barrier when the fetus starts growing rapidly towards the end of the gestation period [37]. Thus, our findings suggest that vascular processes of both the placenta and fetal brain are likely influenced by uterine *Foxa2*. However, further studies are required to confirm this hypothesis.

This study further suggests that genes related to nutrient sensing function of placenta are altered due the absence of *Foxa2* in the uterus. Upon sensing reduced maternal nutrient availability, the placenta converts the maternal tryptophan to produce serotonin or 5-HT (5-hydroxytryptophan) that is necessary for the development of the fetal brain [38]. The IGF-1, mTOR, and AMPK pathways regulate nutrient sensing and signaling in which the placental OGT (O-Linked N-Acetylglucosamine Transferase) acts as a functional integrator of the signaling cascades [39]. Studies show that although AMPK negatively regulates the mTOR pathway, the O-GlcNAcylation of AMPK can lead to higher fetal growth [40]. As an integrator of nutrient sensing machinery, *Ogt* influences placental functions to allocate maternal resources to the growing fetus in a sex-specific manner [14]. This supports the idea that sex-biased growth of the fetus is linked to the sexual dimorphic regulation of nutritional investment by the placenta [41].

In conclusion, the results of our current study indicate that biological roles of uterine *Foxa2* in reproduction and development are more complex and multifaceted than previously thought.

## 4. Materials and Methods

*Mouse model and breeding*. The mouse model used in this study has been described earlier [8]. Floxed *Foxa2* mice [42] were crossed with *Ltf^iCre^* [43] mice to generate conditional knockout animals. Floxed *Foxa2^f/f^* mice (stock no. 022620) and *Ltf^iCre^* mice (stock no. 026030) were obtained from The Jackson Laboratory (Bar Harbor, ME USA). All animal procedures were approved by the Institutional Animal Care and Use Committee of the University of Missouri, and were conducted according to the National Institute of Health Guide for the Care and Use of Laboratory Animals. Timed pregnancy was established by mating of 8-wk old mice by following the littermate breeding strategy described earlier [20]. For *Foxa2* cKO, the dams received i.p. injections of recombinant mouse LIF (10  μg in saline; catalog #554008, BioLegend, San Diego, CA, USA) at 1000 h and 1800 h on GD 3.5 [8]. The day a vaginal plug was observed represented the day 1 of gestation (GD1). The WT and *Foxa2* cKO dams were euthanized on GD15.

*Dissection of placenta and fetus*. The uterine horns were slit opened at the antimesometrial side, and each placentation site with the fetus and its membrane was carefully removed with curved forceps and placed individually into chilled saline using a 12-well culture plate on ice. The metrial gland was peeled off, and the placenta was dissected under a microscope using scissors and curved forceps. Fetuses corresponding to each placentation site were dissected. The weight of each placenta and fetus was recorded. The whole brain was dissected from each fetus. Samples were snap frozen in liquid nitrogen. A portion of the fetal tail was snipped and the sex was determined determination by PCR [44]. Each sample was collected in three biological replicates.

*RNA-seq*. RNA-seq was performed to profile gene expression of the fetal brain and placental samples in genome-wide manner. A total of 24 samples (2 treatments × 2 sexes × 2 tissues × 3 biological replicates) were included in the RNA-seq analysis. Total RNA was isolated from the samples using TRIzol (Catalog 15596026, Thermo Fisher, Waltham, MA, USA). RNA was treated with DNase I to remove genomic DNA and then purified using a RNeasy MinElute Cleanup Kit (QIAGEN, Germantown, MD, USA). RNA quality was checked using a Fragment Analyzer (Advanced Analytical Technologies, Ames, IA, USA). RNA concentration was determined using a Qubit 2.0 Fluorometer (Life Technologies, Carlsbad, CA, USA). Libraries were prepared from total RNA using the Illumina TruSeq kit (San Diego, CA, USA). Each library was sequenced to ~20 million paired end reads. Library preparation and sequencing were performed at Novogene (Sacramento, CA, USA).

*RNA-seq data analysis*. The analysis of RNA-seq data was performed as described in our earlier studies [10,45]. Briefly, the quality of raw sequences was checked with FastQC (Version 0.11.9, Babraham Institute, Cambridge, UK). Trimming of the adaptor sequences was performed by using cutadapt (v2.7, https://cutadapt.readthedocs.io/en/stable/). The *fqtrim* tool (v0.9.7, Johns Hopkins University, Baltimore, MD, USA) was used to perform base quality trimming (Phred score >30) by sliding window scan (6 nucleotides). The quality reads were then mapped to the mouse reference genome GRCm38 using *Hisat2* aligner [46]. The number of reads that mapped to the genes (Ensembl annotation) in each sample was determined from the sequence alignments by using the FeatureCounts tool [47]. The count data was subjected to differential expression analysis (paired-sample) by *edgeR* [48] to identify genes with significant sex-biased expression in the placenta and fetal brain. Hierarchical clustering of gene expression data was performed using the R package *dendextend* [49]. Mutual information analysis was performed using *minet* [50]. The raw and processed data of RNA-seq have been submitted to GEO database (accession # GSE157555).

*Data validation.* We validated the RNA-seq data by two independent methods. First, we compared the expression data of the current study (accession # GSE157555) with the RNA-seq data we generated in a previous study [10] (accession # GSE121799) that used the same strain, same tissues and same gestation time as for the wildtype mice, for a direct comparison. Pearson correlation was performed between expression level of the biological replicates of each gene from the two studies. That analysis showed 96.3% correlation between the two datasets confirming that our RNA-seq data are highly reproducible. Secondly, we performed quantitative real-time PCR (qRT-PCR) analysis with three randomly selected genes, *Kdm5c*, *Camk2b* and *Prl3a1* to validate expression in a subset of samples (fetal brain sample of males and females). The qRT-PCR assays were performed as described in our previous work with *gapdh* as the control gene [10]. A representative qRT-PCR result is shown in Figure S1.

*Functional annotation of differentially expressed genes*. Functional annotation of differentially expressed genes was performed by Gene Ontology (GO) and pathway enrichment analysis. The Ensembl gene IDs were converted to entrez IDs using the R bioconductor package org.Mm.eg.db. The entrez IDs were then used to perform GO and KEGG (Kyoto Encyclopedia of Genes and Genomes) pathway enrichment analysis using ‘goana’ and ‘kegga’ functions respectively, both implemented within *edgeR*. As part of the functional annotation, we also compared the sex-biased genes from our current RNA-seq data with the marker genes of different cell types identified from a previously published single-cell RNA-seq data of mouse placenta and fetal brain [28]. The single-cell RNA-seq count data was downloaded from the Gene Expression Omnibus database (accession numbers GSM2906415, GSM2906465 and GSM2906466), and analyzed using the R package *Seurat* to perform a canonical correlation analysis (CCA) [51]. The purpose of this analysis was to identify the cell types in which gene expression varied in a canonical correlation manner between the placenta and fetal brain. By identifying integration anchors for the first 20 dimensions of data variation, the placenta and fetal brain data were integrated and the integrated data was subjected to principal component analysis (PCA) by applying non-linear dimensional reduction methods UMAP (Uniform Manifold Approximation and Projection) and tSNE (t-distributed stochastic neighbor embedding) [52]. After identifying the clusters of cell types, the ‘FindAllMarkers’ function was used to identify the marker genes of the predicted cell clusters. The endothelial cell cluster of the placenta and fetal brain was identified based on cell-specific marker genes available in the *PanglaoDB* [53].

## Data Availability

All the raw and processed data of this study have been submitted to the Gene Expression Omnibus database under the accession number GSE157555.

## Supplementary Materials

The following are available online at https://www.mdpi.com/article/10.3390/ijms22189693/s1, Figure S1: qRT-PCR data for *Kdm5c* gene. The graph shows the plot of relative fluorescence unit (RFU) of samples (three) with *Kdm5c* (test) and *gapdh* (control) genes (A). The raw CT (cycle threshold) values are shown in B. Table S1: List of genes expressed in the brain and placenta in sex-biased manner in the WT mice. The mean expression (read count) of each gene is provided for fetal brain and placenta of both the sexes. Brain F/m, Brain M/f, Placenta F/m, and Placenta M/f are the four types of the observed sex-biased gene expression. The fetal sex is indicated in upper case if the gene was expressed at a higher level in that sex relative to the other, Table S2: List of genes expressed in the brain and placenta in sex-biased manner in the *Foxa2* cKO mice. The mean expression (read count) is also provided for fetal brain and placenta of both the sexes. Brain F/m, Brain M/f, Placenta F/m, and Placenta M/f are the four groups or modules of sex-biased gene expression. The fetal sex is indicated in upper case if genes were expressed at a higher level in that sex relative to the other, Table S3: The list of genes exhibiting sexually conflicting expression between WT and *Foxa2* cKO mice, Table S4: List of sex-biased genes associated with nutrient sensing and growth regulation functions., Table S5: List of longevity genes expressed in the fetal brain and placenta. The mean expression of these genes in WT and cKO fetal brain and placenta of both sexes are shown, Table S6: List of angiogenesis-related genes, and expression in brain and placenta of WT and cKO mice, Table S7: List of marker genes of the vascular endothelial cells identified from the published single-cell RNA-seq data of mouse placenta and fetal brain. The sex-biased expression of those genes in the bulk RNA-seq data (current study) is also shown.
