# Supplementary figures and images for: Fetal Brain Elicits Sexually Conflicting Transcriptional Response to the Ablation of Uterine Forkhead Box A2 (Foxa2) in Mice
Source: Int J Mol Sci. 2021 Sep 7;22(18):9693. doi: 10.3390/ijms22189693 (PMC8468108; doi:10.3390/ijms22189693)

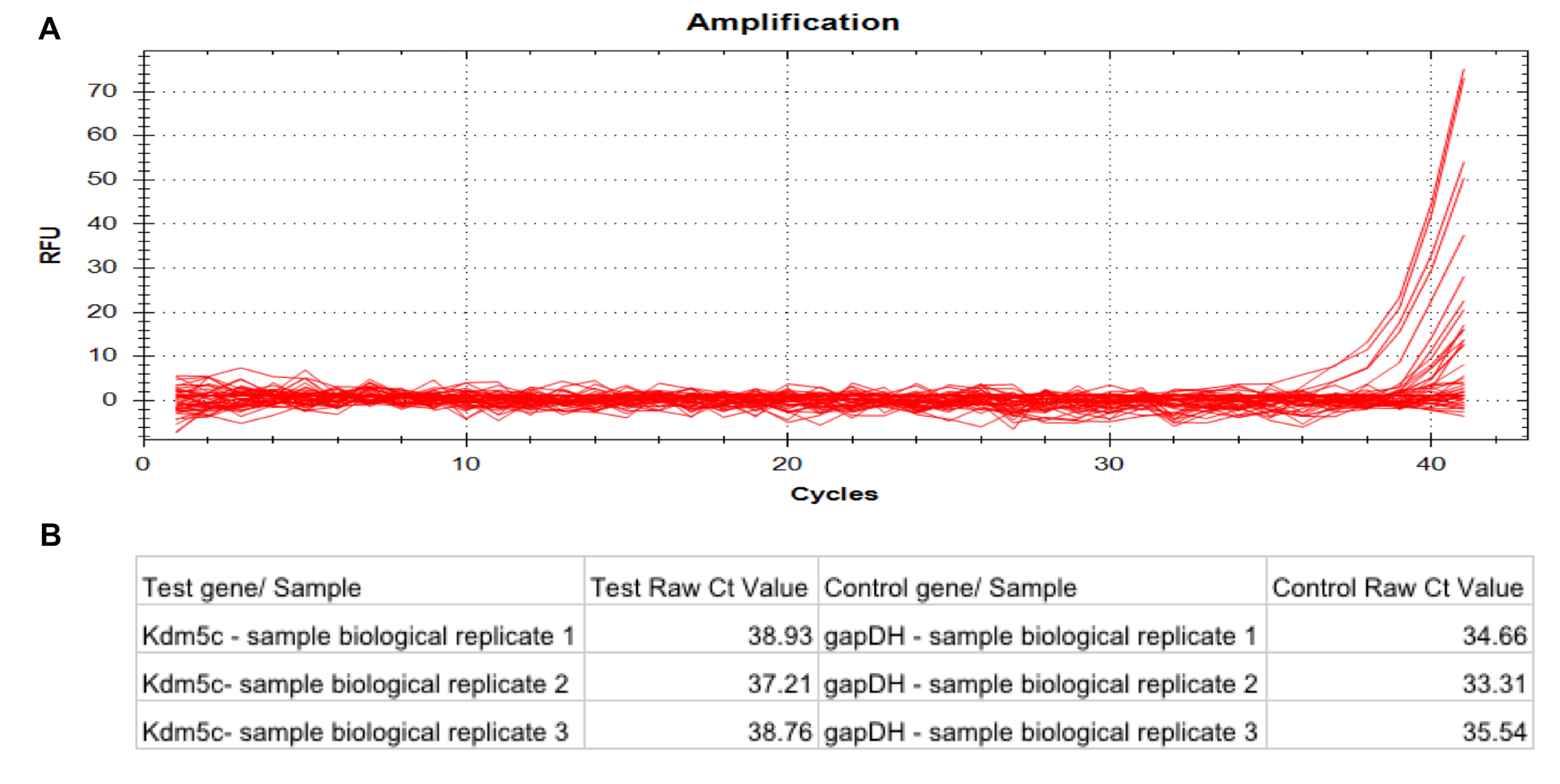

Supplement: Supplementary file 1 [file ijms-22-09693-s001.zip › Supplementary Figure S1.tiff]
